# Viability test of fish scale collagen (*Oshpronemus gouramy*) on baby hamster kidney fibroblasts-21 fibroblast cell culture

**DOI:** 10.14202/vetworld.2018.506-510

**Published:** 2018-04-18

**Authors:** Chiquita Prahasanti, Denny Tri Wulandari, Noer Ulfa

**Affiliations:** Department of Periodontic, Faculty of Dental Medicine, Universitas Airlangga, Surabaya, Indonesia

**Keywords:** bone graft, collagen, gouramy fish scale, viability, 3-(4.5-dimethylthiazole-2-yl)2.5-diphenyl tetrazolium bromide

## Abstract

**Aim:**

This study aims to examine the toxicity of collagen extracted from gouramy fish scales (*Oshpronemus gouramy*) by evaluating its viability against baby hamster kidney fibroblasts-21.

**Materials and Methods:**

Collagen was extracted from gouramy fish scales (*O. gouramy*) with 6% acetic acid. Its results were analyzed using Fourier-transform infrared spectroscopy and freeze-dried technique. Its morphology then was analyzed with scanning electron microscope. Afterward, 3-(4.5-dimethylthiazole-2-yl)2.5-diphenyl tetrazolium bromide assay was conducted to compare cells with and without fish scale collagen treatment.

**Results:**

Collagen extracted from gouramy fish scales had no influence statistically on cultured fibroblast cells with a statistical significance (2-tailed) value of0.754 (p>00025).

**Conclusion:**

Collagen extracted from gouramy fish scales has high viability against BHK21 fibroblast cells.

## Introduction

Periodontal disease is a chronic bacterial infection characterized by persistent chronic inflammation, connective tissue damage, and bone destruction [1]. The bone damage that accompanies periodontal disease varies greatly. Thus, the same patient in different areas can have different damage types. As a result, periodontal treatments needed to deal with the tissue destruction also vary [2]. The periodontal treatments have changed over three decades from resective to regenerative. The regenerative technique requires bone grafts, bioactive molecules, and membranes [3].

Bone grafts have been used extensively to stimulate the new bone formation and periodontium tissue regeneration, functioning as a structural scaffold and matrix for attachment and proliferation of anchorage-dependent osteoblasts [3,4]. The gold standard for bone grafts is autograft. Unfortunately, autograft has some limitations such as lack of transplant materials availability, donor morbidity, inflammation, and bone resorption. An allograft or synthetic grafting material can overcome the limitations of autograft even though both alternatives still have limitations in both immunogenicity and osteoinductivity [5]. Similarly, xenografts such as bovine bone have widely been used, but the transmission of disease from cattle, especially spongiform encephalopathy leads to a very urgent need for an alternative bone graft. Xenografts have several main advantages such as osteoconductive ability and many available raw materials. One of the xenograft materials used as bone grafts is type I collagen, considered as a gold standard in periodontal tissue regeneration [6].

Various alternative sources of collagen have already been used for scaffolds such as freshwater and seawater fish’s scale, chicken’s shank, as well as sea animals (squid, octopus, jellyfish, and starfish). However, collagen extracted from fish is safer. Unfortunately, the use of fish is usually only on their meat while their bone and scales are considered as wastes although their skin, bones, and scales also have the potential to be extracted into collagen. An *in vitro* research on the cytotoxicity of collagen extracted from freshwater fish scales, namely, *Labeo rohita* (Rohu) and *Catla catla* (Catla), conducted by Pati *et al*. [7] revealed that the collagen extracted from those freshwater fish scales could stimulate cell life without generating significant cytotoxic effects. Similarly, another previous research conducted by Zhang showed that gouramy fish scales could be used as a collagen source, especially type 1 collagen. Fish scale is biocomposite with high type 1 collagen fiber and hydroxyapatite Ca10(OH)2(PO4)6 [8].

Indonesia has great potential as a producer of collagen extracted from fish because of its vast territorial waters and abundant fishery resources. In addition, Indonesia’s sea and river also have abundant natural resources such as many fish species. For instance, gouramy fish come from the waters of the Sunda West Java region. Gouramy fish are considered as the best freshwater fish species in terms of high demand and price stability [9,10].

Consequently, collagen extracted from gouramy fish scales can be assumed as an alternative raw material for bone grafts. In the dentistry field, bone graft materials have been used in the oral cavity, nevertheless, must be biocompatible. It means that bone graft materials can be accepted by the host, non-toxic, irritant, non-carcinogenic, and non-allergen [11].

Thus, cytotoxicity test as a part of the evaluation in dentistry is required as a standard screening procedure. One of the methods for assessing the cytotoxicity of an ingredient is using a reagent, 3-(4.5-dimethylthiazole-2-yl)2.5-diphenyl tetrazolium bromide (MTT assay) [12]. MTT assay is often used as the golden standard of biocompatibility test. MTT assay aimed to measure cytotoxic effects of scaffolds on cells as an indicator of cell proliferation rates. In other words, the ability of cells to proliferate and develop in scaffolds plays a role as a direct indicator of the cytotoxic effects of the scaffolds [13]. Therefore, this research aimed to determine the viability of collagen extract derived from gouramy fish scales on baby hamster kidney fibroblasts-21 (BHK21) cell culture.

## Materials and Methods

### Ethical approval

This research was an experimental laboratory study and received ethical clearance from the Bioethics Committee of Faculty of Dental Medicine, Universitas Airlangga, Surabaya, with number 213/KKEPK.FKG/XII/2015.

### Collagen extracted from gouramy fish scales

Gouramy fish scales were extracted into collagen with 6% acetic acid. The extracts then were analyzed with Fourier-transform infrared spectroscopy (FTIR) to ensure whether the extracts obtained were type I collagen. Scanning electron microscope (SEM) analysis was performed to observe their morphology.

### MTT test on BHK21 fibroblast cell culture

The MTT test was performed using BHK21 fibroblast cells. The extraction of gouramy fish scales into collagen was conducted at the Laboratory of Faculty of Veterinary Medicine, Universitas Airlangga. Meanwhile, the FTIR and SEM analysis were carried out at the Laboratory of FTI Institut Teknologi Sepuluh Nopember, Surabaya. MTT assay performed in accordance with the procedures of the PUSVETMA laboratory.

Afterward, samples used in this research were divided into two groups, cells treated with collagen extracted from gouramy fish scales were treatment group and cells without collagen extracted from gouramy fish scales were control group. Viability, the number of living cells, then was observed by calculating CD50 with MTT assay (3-(4,5-dimethylthiazole-2-yl)-2,5-diphenyltetrazolium bromide. Their absorbance indicated with a purplish-blue portion of formazan was read using the enzyme-linked immunosorbent assay (ELISA) reader with a wavelength of 550-620 nm. The percentage of live cells was measured using the following formula:


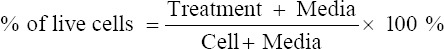


Note:

% of live cells = Percentage of live cell count after the test
Treatment = Optical density value of formazan in each sample after the test
Media = Optical density value of formazan on media
Cell = Optical density value of formazan on control cells.
Control cell as a positive control in the culture medium is considered to have a live cell percentage of 100%.

### Statistical analysis

The data obtained were analyzed statistically using t-test with 95% confidence level. The data obtained were tested using a normality test, Shapiro–Wilk. The homogeneity of the research data was tested using Levene test.

## Results

### Collagen extracted from gouramy fish scales

**Figure-1:**
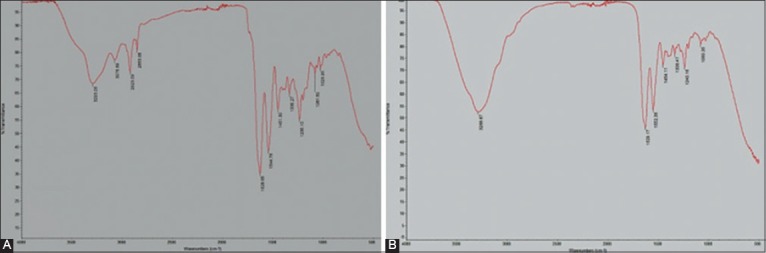
Fourier Transform Infrared Spectroscopy result; A. Collagen Fleece; B. Collagen extract derived from Gouramy fish scale.

Patent collagen fleece was used as guidance for FTIR analysis in ensuring the type of collagen extracted from gouramy fish scales. Results of the FTIR analysis showed that the composition and functional structure of those cells in the group treated with collagen extracted from gouramy fish scales were similar to patent collagen fleece (Figure-1 and Table-1). Gouramy fish scales had p=0.945. In other words, both groups had p>0.05. Thus, it can be said the data obtained were normally distributed.

**Table-1:** Composition of collagen fleece^®^ with collagen extracted from gouramy fish scales.

Collagen fleece^®^	Collagen extracted from gouramy fish scales
Poly (6-methylcaprolactam)	Polyamide 6+polyamide 6,6
Silk II	Poly (6-methylcaprolactam)
Silk I	Poly (4-methylcaprolactam)
Polyamide 6+polyamide 6,6	Poly (5-methylcaprolactam)
Polymer phthalimide	Poly (3-methylcaprolactam)
Poly (4-methylcaprolactam)	Silk II
Poly (5-methylcaprolactam)	Poly (n-methyl acrylamide)
Grilamid TR 55	Grilon TR 27
Poly (3-methylcaprolactam)	Polyamide 6

### MTT test on BHK21 fibroblast cell culture

Well plates of BHK21 fibroblast cells appeared purplish similar to the color of the control cells after the administration of dimethyl sulfoxide (DMSO).

### Statistical analysis

Results of the Shapiro–Wilk test showed that those live cells of BHK21 without collagen extracted from gouramy fish scales had p=0.333. Moreover, results of the Levene test indicated p=0.161 (p>0.05). It means that the data of both groups were homogeneous. Since the data were homogeneous and normally distributed, the hypothesis test was statistically performed using a parametric statistic test. Independent-sample t-test was used to analyze the differences of the mean live BHK21 cell count between the control and treatment groups with 95% confidence level (α=0.05) (Table-2).

**Table-2:** Results of independent-sampale t-test (The mean optical density values of those two groups).

Groups	n	Mean	Cell viability (%)	Sig. (2-tailed)
Control group	8	0.44638	100	0.754
Treatment group	8	0.41850	94	

p≥0.025=No significant difference

## Discussion

Collagen extraction was performed in an acidic environment. The acid pH (4-5) is isoelectric for the non-collagen protein components so it will be easily coagulated and removed. Inflammation process at the time of immersion with 6% acetic acid began on the day 7 caused by the water penetration into fish scales and an increase in H^+^ ions triggered by the acetic acid. At this stage, collagen is not fully soluble due to low collagen solubility. Sterilization of collagen extract was conducted using gamma-ray radiation after freeze-drying. Gamma-ray radiation is considered as a physically useful cross-linking method to stabilize collagen without cytotoxic potential. Another advantage of gamma radiation, its process is relatively fast, and no residuals remain [14,15].

The results of the FTIR analysis qualitatively showed that the position of Amide I was at a distance between 1600 and 1700/cm. Amide I corresponds to the vibration of the carbonyl group strain (C=O) in the polypeptide. This amide is a sensitive marker of the secondary peptide structure. Meanwhile, Amide II is normally located at a distance of 1550-1600/cm. Amide II is used as an indicator of hydrogen bonds in collagen triple helix. Amide II associated with N-H vibration bonds and C-N strain. According to the literature, Amide III also corresponds to the C-N strain and N-H deformation ranging from 1240/cm [16].The position of Amide A corresponds to the frequency of the N-H strain [16,17].

In this research, NH vibration strain occurred at a distance of between 3400 and 3440/cm [7,16,17].The results of FTIR also indicated that the presences of Amide I, Amide II, and Amide III were located at 1629.17/cm, 1552.38/cm, and 1240.16/cm. The results of the FTIR analysis confirmed that signals derived from Amide I and Amide II indicating collagen extracted from gouramy fish scales are dominated by a helical structure. The secondary structural integrity of the type 1 collagen fibers then was determined by a ratio of Amide III and Amide II (1240.16/cm/1552.38/cm) reaching 1 [15,16]. Therefore, collagen extracted from gouramy fish scales used in this research was categorized into type 1 collagen that can be used as a scaffold in bone tissue engineering. This is similar to previous research conducted by Zheng *et al*. [18], suggesting that type 1 collagen nanofibrous scaffold can support the growth of mesenchymal stem cells and be used for bone tissue engineering.

Based on the results of the SEM morphology analysis, collagen extracted from gouramy fish scales looked like fibers interconnected (meshwork) with pores among them (191.6-385.3 m), similar to collagen extracted from other freshwater fish (Rohu and Catla) ever observed by Pati *et al*. [7]. The optimum size of pores for bone regeneration is 100-500 μm. The porosity size is important because the porosity affects how many cells, materials, and nutrients can penetrate and grow in scaffolds used to perform cell hatchery, cell migration, matrix deposition, vascularization, as well as transport of nutrients to and from cells. The size of graft particles if it is too small, the graft quickly resorbed by macrophages so that its ability to induce bone is not optimal. In contrast to graft with a particle size too large, resorption by macrophages is too slow to block the process of new bone formation [19-21].

In the MTT assay, moreover, cultured BHK21 fibroblast cells were used because of several reasons; first, their passage can be 50-70 times; second, their cell growth rate is high; and third, their cell integrity can be maintained. BHK21 has been widely used in dentistry field for the viability test [22]. MTT assay is absorbed into living cells then broken down through a reduction reaction by reductase enzymes in the mitochondrial respiration chain into formazan soluble in a purple solvent. DMSO is added to stop the enzymatic reaction and dissolve the formazan so that the purple color of the formazan can be read its absorbance spectrophotometrically with ELISA reader. The absorbance represents the number of living cells. The stronger the purple color intensity is, the higher the absorbance will be. In other words, this suggests that the more MTT assay is absorbed into living cells can lead to more formazan formation. Consequently, this absorbance can be used to calculate the percentage of living cells as cell response [23].

In addition, a substance, according to Telli *et al*. [24], on the toxicity parameters of CD50, can be said to be toxic if the percentage of living cells after exposure to the substance is <50%. In this research, the viability percentage of fibroblasts after the administration of collagen extracted from gouramy fish scales was 94%, higher than 50%. As a result, the collagen extracted from gouramy fish scales can be considered as a non-toxic substance.

Statistically, there was no significant difference in the mean of live BHK21 fibroblast cell count between in the group treated with collagen extracted from gouramy fish scales and the group without collagen extracted from gouramy fish scales with p=0.754 (p>0.25 - 2 tailed). In other words, type 1 collagen extracted from gouramy fish scales can influence cell growth indicated with MTT assay. Similarly, two previous researchers conducted by Pati *et al*. [7] and Leong *et al*. [25] revealed that type 1 collagen from freshwater fish could increase the fibroblast cells proliferation so that the collagen extracted from gouramy fish scales is non-toxic and can be used as a collagen-based bone graft potential candidate.

## Conclusion

Collagen extracted from gouramy fish scales has high viability against BHK21 fibroblast cells culture. As a result, collagen extracted from gouramy fish scales can be used as an alternative biomaterial of bone grafts needed to optimize bone defect treatments.

## Authors’ Contributions

CP: Conception and design of the study. DTW: Acquisition of data. DTW and NU: Analysis and interpretation of the data. CP, DTW, and NU: Drafting and revising the manuscript critically for important intellectual content. All authors have read and approved the final manuscript.

## References

[1] Newman M, Takei H, Klokkevold P, Carranza F (2012). Carranza's Clinical Periodontology.

[2] Cochran D.L (2008). Inflammation and bone loss in periodontal disease. J. Periodontol.

[3] Siaili M, Chatzopoulou D, Gillam D.G (2012). An introduction to periodontal regeneration. Dent. Nurs.

[4] Dumitrescu A.L (2011). Bone grafts and bone grafts substitutes in periodontal therapy.

[5] Jimi E, Hirata S, Osawa K, Terashita M, Kitamura C, Fukushima H (2012). The current and future therapies of bone regeneration to repair bone defects. Int. J. Dent.

[6] Parenteau-Bareil R, Gauvin R, Berthod F (2010). Collagen-based biomaterials for tissue engineering applications. Materials (Basel).

[7] Pati F, Datta P, Adhikari B, Dhara S, Ghosh K, Mohapatra P (2012). Collagen scaffolds derived from freshwater fish origin and their biocompatibility. J Biomed Mater Res Part A.

[8] Zhang F, Wang A, Li Z, He S, Shao L (2011). Preparation and characterisation of collagen from freshwater fish scales. Food Nutr. Sci.

[9] Him (2007). Gurami Masih Unggul (Gouramy is still superior).

[10] Kasim S (2013). The effect of acid solvent variation on collagen extraction from stingray (*Himantura gerrardi*) and tuna (*Thunnus* sp). Pharm. Pharmacol. J.

[11] Yuliati A (2005). Viabilitas sel fibroblas BHK-21 pada permukaan resin akrilik rapid heat cured (Viability of fibroblast BHK-21 cells to the surface of rapid heat cured acrylic resins). Dent. J. (Majalah Kedokt Gigi).

[12] Siregar F, Hadijono B.S (2000). Uji Sitotoksisitas dengan Esei MTT (Cytotoxicity Test with an MTT Assay). J. Kedokt. Gigi. UI.

[13] Bharatham B.H, Bakar M.Z.A, Perimal E.K, Yusof L.M, Hamid M (2014). Development and characterization of novel porous 3d alginate-cockle shell powder nanobiocomposite bone scaffold. Biomed. Res. Int.

[14] Yamada S, Yamamoto K, Ikeda T, Yanagiguchi K, Hayashi Y (2014). Potency of fish collagen as a scaffold for regenerative medicine. Biomed. Res. Int.

[15] Erizal E, Perkasa D.P, Abbas B (2013). Synthesis of fish scales gelatin-chitosan crosslinked films by gamma irradiation techniques. Sci. J. Appl. Isot. Radiat.

[16] Júnior Z.S.S, Botta S.B, Ana P.A, França C.M, Fernandes K.P.S, Mesquita-Ferrari R.A (2015). Effect of papain-based gel on Type I collagen--spectroscopy applied for microstructural analysis. Sci. Rep.

[17] Kiew P.L, Mat Don M (2013). The influence of acetic acid concentration on the extractability of collagen from the skin of *Hybrid clarias* sp. and its physicochemical properties : A preliminary study. Focus Mod. Food Ind.

[18] Zheng W, Zhang W, Jiang X (2010). Biomimetic collagen nanofibrous materials for bone tissue engineering. Adv. Eng. Mater.

[19] Alvarez K, Nakajima H (2009). Metallic scaffolds for bone regeneration. Materials (Basel).

[20] da Cruz A.C.C, Pochapski M.T, Daher J.B, da Silva J.C.Z, Pilatti G.L, Santos F.A (2006). Physico-chemical characterization and biocompatibility evaluation of hydroxyapatites. J. Oral. Sci.

[21] Larjava H (2012). Oral Wound Healing.

[22] Meizarini A (2005). Cytotoxicity of the cyanoacrylate restoration material with variation of powder and liquid ratio by using MTT assay. Dent. J. (Majalah Kedokt Gigi).

[23] Wijaya J, Salenussa J, Marantika J (2013). Potential of lime leaf methanol extract (*Harmsiopanax aculeatus* Harms) as antimalarial drugs. Prog. Kreat Mhs Penelit.

[24] Telli C, Serper A, Dogan A.L, Guc D (1999). Evaluation of the cytotoxicity of calcium phosphate root canal sealers by MTT assay. J. Endod.

[25] Leong L.M, Sahalan A.Z, Tan L.H, Mustafa N.H, Rajab N.F (2015). *Clarias batrachus* collagen extract increases fibroblast cell adhesion, migration and proliferation. J. Appl. Pharm. Sci.

